# Prospective Study of the Quality of Colonoscopies Performed by Primary Care Physicians: The Alberta Primary Care Endoscopy (APC-Endo) Study

**DOI:** 10.1371/journal.pone.0067017

**Published:** 2013-06-27

**Authors:** Michael R. Kolber, Clarence K. W. Wong, Richard N. Fedorak, Brian H. Rowe, on behalf of the APC-Endo Study Physicians

**Affiliations:** 1 Department of Family Medicine, University of Alberta, Edmonton, Alberta, Canada; 2 Department of Gastroenterology, University of Alberta, Edmonton, Alberta, Canada; 3 Department of Emergency Medicine, University of Alberta, Edmonton, Alberta, Canada; Copenhagen University Hospital Gentofte, Denmark

## Abstract

**Background:**

The quality of colonoscopies performed by primary care physicians (PCPs) is unknown.

**Objective:**

To determine whether PCP colonoscopists achieve colonoscopy quality benchmarks, and patient satisfaction with having their colonoscopy performed by a primary care physician.

**Design:**

Prospective multi-center, multi-physician observational study. Colonoscopic quality data collection occurred via completion of case report forms and pathological confirmation of lesions. Patient satisfaction was captured by a telephone survey.

**Setting:**

Thirteen rural and suburban hospitals in Alberta, Canada.

**Measurements:**

Proportion of successful cecal intubations, average number of adenomas detected per colonoscopy, proportion of patients with at least one adenoma, and serious adverse event rates; patient satisfaction with their wait time and procedure, as well as willingness to have a repeat colonoscopy performed by their primary care endoscopist.

**Results:**

In the two-month study period, 10 study physicians performed 577 colonoscopies. The overall adjusted proportion of successful cecal intubations was 96.5% (95% CI 94.6–97.8), and all physicians achieved the adjusted cecal intubation target of ≥90%. The average number of ademonas detected per colonoscopy was 0.62 (95% CI 0.5–0.74). 46.4% (95% CI 38.5–54.3) of males and 30.2% (95% CI 22.3–38.2) of females ≥50 years of age having their first colonoscopy, had at least one adenoma. Four serious adverse events occurred (three post polypectomy bleeds and one perforation) and 99.3% of patients were willing to have a repeat colonoscopy performed by their primary care colonoscopist.

**Limitations:**

Two-month study length and non-universal participation by Alberta primary care endoscopists.

**Conclusions:**

Primary care physician colonoscopists can achieve quality benchmarks in colonoscopy. Training additional primary care physicians in endoscopy may improve patient access and decrease endoscopic wait times, especially in rural settings.

## Introduction

Colorectal cancer is the second most common cause of death from a malignancy in Canada [1], [2]. Colonoscopy is a cost-effective tool in screening patients for colorectal cancer [3], [4], and is also used to investigate patients with gastrointestinal symptoms [5].

Colorectal cancer screening campaigns have fueled an increased demand for colonoscopies [6]–[8], while a shortage of colonoscopists has contributed to excessive wait times [9], [10]. In Canada, endoscopic wait times, depending on the indication, are up to 7.2 times longer than recommended targets [10]. For example, only 41% of patients with a positive fecal occult blood test had a colonoscopy within recommended timeframes [10], [11]. In Canada, gastroenterologists and general surgeons perform 97% of all colonoscopies [12]. Training primary care physicians (PCPs) in gastrointestinal medicine and endoscopy may improve patient access and wait times for endoscopy, especially for rural patients.

Although recent studies demonstrate that primary care physicians can perform quality colonoscopies [13]–[15], earlier research showed less optimal results [16]–[18]. In addition, many studies had methodological issues including single endoscopist reporting [13]–[21], retrospective data collection [13]–[16], [19]–[24], or the use of older monocular endoscopes [16]–[20]. Other studies did not report the method [18], [19], [21] or potentially used inaccurate methods of confirming cecal intubation [16], [20], [25]. A systematic review, that concluded that primary care physicians can perform quality colonoscopies [26], was criticized [27] for including a study where an on-site gastroenterologist could assist the PCP colonoscopist [28]. Recent Canadian studies, using administrative databases, claim future cancer rates are higher when colonoscopies are performed by non-gastroenterologists [29]–[31]. Clearly, additional high quality research is required to determine whether primary care physicians can perform quality colonoscopies.

The Alberta Primary Care Endoscopy (APC-Endo) Study is the first Canadian, prospective, multi-center health outcomes study to examine the quality of colonoscopic procedures performed by a group of primary care physicians in Canada. The study’s primary objective was to determine the proportion of successful cecal intubations and adenomas detected in colonoscopies performed by Alberta family physicians and general internists. These results were compared to standards defined by the United States Multi-Society Task Force on Colorectal Cancer (USMSTF) [32], [33]. Secondary objectives included a determination of serious adverse events (SAEs), other quality markers such as procedural and withdrawal times, patient comfort level during the procedure, and the percentage of patients referred to a specialist for their gastrointestinal problem, and patient satisfaction related to the colonoscopy.

## Methods

All 17 family physicians and general internists performing colonoscopies in Alberta were identified and approached to voluntarily participate in the study. A pre-study questionnaire was sent to participating physicians to explore their practice characteristics and colonoscopic experience. Both participating physicians and their assistants completed case report forms at the time of the patients’ colonoscopy (Form S1). Prior to having their colonoscopy, patients provided written consent to a patient satisfaction telephone survey adapted from the Group Health Association of America 9 (GHAA-9) [34] and the University of Calgary Gastrointestinal Endoscopy Unit Patient Satisfaction Questionnaires [35] (Form S2). Patients were excluded from participating in the telephone survey if they were under 18 years of age, likely unable to be contacted for the survey, did not comprehend or speak English, or were cognitively impaired rendering them unable to complete the initial consent for their colonoscopy. Centrally located study assistants administered the patient satisfaction telephone survey approximately one month after the patient’s colonoscopy**.** All significant lesions required pathological confirmation to be included in the analysis and independent external adjudicators reviewed all potentially serious adverse events.

### Ethics Statement

Ethical approval for the study was granted by the University of Alberta’s Health Research Ethics Board.

### Outcome Measures

#### Proportion of successful cecal intubations

Colonoscopy completion was determined by visualization of any cecal or ileal landmarks including the appendiceal orifice, cecal trifolds, ileocecal valve or intubation of the terminal ileum. The crude proportion of successful cecal intubations was calculated by dividing the total number of colonoscopies where cecal intubation was achieved by the total number of colonoscopies attempted. The adjusted proportion of successful cecal intubations was calculated by dividing the total number of colonoscopies where cecal intubation was achieved by the total number of colonoscopies attempted minus the number of procedures limited by poor bowel preparation, colonic stricture, equipment failure or severe endoscopic colitis in which forward advancement was not possible.
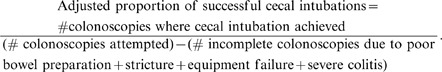



As all physicians performed both diagnostic and screening colonoscopies, competency in cecal intubation was determined by comparing the adjusted proportion of successful cecal intubations to the USMSTF targets of ≥90% [32], [33].

#### Adenoma detection

The average number of adenomas detected per colonoscopy was determined by dividing the total number of pathologically confirmed adenomas by the number of colonoscopies performed. In addition, the proportion of patients ≥50 years, having their first colonoscopy with at least one pathologically confirmed adenoma was compared to USMSTF benchmarks of 25% for males and 15% for females [32], [33]. Advanced adenomas were defined as adenomas greater than 1cm, or containing villous components or high-grade dysplasia on pathology. Serrated adenomas, an evolving pathological entity of polyps initially thought to be hyperplastic, but now shown to have distinct pathological architecture and potential for dysplasia [36] were grouped with adenomas for analysis.

#### Potentially serious adverse events

Potentially serious adverse events (SAEs), reported by physicians or patients, were investigated and externally adjudicated by two independent adjudicators. A third independent adjudicator (a gastroenterologist) reconciled any adjudicator disagreements. Definitions of serious adverse events were derived from the American Society of Gastrointestinal Endoscopy (ASGE) [37], [38]. Bleeding was defined as blood loss, resulting in admission to hospital, a blood transfusion, a second colonoscopy or surgery. Perforations required clinical and radiographic evidence. Procedural sedation adverse events were defined as occurring if the colonoscopy is prematurely aborted, reversal agents are required, the patient requires assisted ventilation, or the patient is admitted to hospital after the procedure for any new cardiac or respiratory condition related to use of sedation agents. Frequency of SAEs were compared to published standards of bleeding (<1/100) [32], [33], perforation (1/500 to 1/1000) [33], and procedural sedation (<1/100) [32].

#### Statistical analysis

Prior to commencing the study, it was estimated that approximately 15 primary care physicians perform colonoscopies in Alberta. A convenience sample of three primary care colonoscopists revealed they perform an average of 20 colonoscopies per month. Assuming that 12 of the 15 physicians would agree to participate, approximately 240 procedures would be performed per month. Therefore, for our two-month study, it was estimated that 480–500 cases would be available to analyze. Assuming that 80% of patients would agree to the post-procedure telephone survey, 384 phone interviews would be performed. Estimating an overall adjusted cecal intubation rate of 90%, this sample size would provide 2.5% confidence intervals (CI) around the main point estimates. A significantly larger sample size would be required to provide CI within +/−1%, which was not feasible for the primary care colonoscopists and given funding constraints.

Binary outcomes, such as proportion of successful cecal intubations or age- and sex-specific average number of adenomas detected, are reported as percentages with 95% confidence intervals (CI) and compared to quality standards using z statistics. Continuous variables are reported as means and standard deviations (SD) or medians and interquartile ranges (IQR), as appropriate. Logistic regression analysis was performed to determine which variables predicted incomplete colonoscopies; results are reported as odds ratio (OR) with 95% CI. Statistical analysis was performed with Stata™ 11.

## Results

Ten of 17 identified primary care colonoscopists (eight family physicians and two general internists) participated in the study. These physicians had performed an estimated median of 1850 colonoscopies (interquartile range [IQR] 1400–4000) in their career prior to commencing the study. All physicians perform polypectomies, and nine out of 10 administer their own procedural sedation. They perform their colonoscopies at 13 different hospitals (i.e., three physicians perform endoscopy at two sites), including 11 rural sites. Only three sites have local surgical back up (Table 1).

**Table 1:** Participating physician and practice characteristics for the Alberta Primary Care Endoscopy study.

Physician	PhysicianGender	Physician Group	Location	Number of Colonoscopies in Practice	Years Performing Colonoscopies	PerformPolypectomies	Sedation Administration	Local Surgical Backup
1	M	FM	Rural	1700	10	Yes	Self	No
2	M	FM	Rural	4000	10	Yes	Self^a^	No
3	M	FM	Rural	8000	21	Yes	Self	No
4	M	GIM	Regional	2800	14	Yes	Anaesthesia	Yes
5	F	GIM	Regional	60	1	Yes	Self	Yes
6	M	FM	Rural	1500	6	Yes	Self	No
7	M	FM	Rural	2000	15	Yes	Self	No
8	M	FM	Rural	4000	15	Yes	Self^a^	Yes
9	M	FM	Rural	1400	4	Yes	Self	No
10	M	FM	Rural	1000	3	Yes	Self	No
**Totals**	**90% M**	**80% FM**	**80% Rural**	**Median: 1850**	**Median: 10**	**100%**	**90% Self**	**30%**

M = male; F = female.

FM = Family Medicine; GIM = General Internal Medicine.

a General practice – Anaesthetists (Gp-A).

Data collection occurred between March and August 2010. Ten physicians performed a total of 579 colonoscopies during the study using combinations of Olympus 160 and 180 SD and 180 HD as well as Pentax 70 SD and 90i series HD colonoscopes. Two patients, under 18 years of age, were excluded, leaving 577 colonoscopies for analysis. Each physician performed a median of 52 colonoscopies (IQR 38–78) in their two-month study period. The mean patient age was 57.6 years of age (SD: 13.3) and 51% were female (Table 2). For 65% of patients, the study colonoscopy was their first colonoscopy. All study physicians performed both screening and diagnostic colonoscopies, with 45.9% of the colonoscopies performed to screen for colorectal cancer (including family history of colorectal cancer, Lynch syndrome or familial adenomatous polyposis; positive FOBT; and average risk screening) and 40.2% were performed to investigate gastrointestinal symptoms.

**Table 2:** Patient characteristics for the Alberta Primary Care Endoscopy study.

Physician	Colonoscopies Performed	Mean Patient Age (years)	Female (%)	Inpatient (%)	First-timeColon (%)	Indications (%)	
						Screening	Symptoms
1	54	56.4	59.3	5.6	51.9	37.0	42.6
2	72	57.0	52.8	2.8	66.7	48.6	36.1
3	95	59.3	59.0	0.0	66.3	63.2	26.3
4	31	60.8	32.3	0.0	64.5	38.7	41.9
5	38	47.3	42.1	2.6	68.4	29.0	47.4
6	40	57.8	52.5	0.0	62.5	52.5	35.0
7	38	58.3	57.9	0.0	73.7	36.8	52.6
8	82	61.5	45.1	2.4	59.8	42.7	47.6
9	78	59.4	46.2	1.3	69.2	57.7	30.8
10	49	52.3	55.1	4.1	69.4	24.5	61.2
**Overall Totals/Means**	**577**	**57.6 (SD = 13.3)**	**51.1 (47.0, 55.2)**	**1.9 (0.7, 3.2)**	**65.0 (61.1, 69.0)**	**45.9 (41.8, 50.0)**	**40.2** **(36.1, 44.3)**

% = percentage; SD = standard deviation.

Overall totals/means : reported with 95% confidence intervals except age reported with standard deviations.

Screening includes : family history of colorectal cancer, Lynch syndrome or familial adenomatous polyposis; positive FOBT; and average risk screening.

### Outcomes

#### Cecal intubation

Cecal intubation was achieved in 550 of 577 colonoscopies for a crude proportion of successful cecal intubations of 95.3% (95% CI 93.3–96.9) (Table 3), which was statistically greater than 90% (p = 0.00004). The terminal ileum was intubated in 41.3% (95% CI 36.9–45.1) of completed colonoscopies and 89.3% of successful cecal intubations reported visualizing two or more cecal landmarks or the terminal ileum. Individual physician’s crude proportion of successful cecal intubations ranged from 87.8–100%. After seven cases were excluded (stricture/obstruction [four], poor bowel preparation [two] and severe inflammatory bowel disease [one]), the overall adjusted proportion of successful cecal intubations was 96.5% (95% CI 94.6–97.8). All 10 physicians achieved the adjusted cecal intubation target of ≥90%. Using the USMSTF definition of adjusted cecal intubation (only excluding poor preparation and severe colitis), the group’s adjusted proportion of successful cecal intubations was 95.8% (95% CI 93.8–97.3) and only one physician’s achieved less than 90%.

**Table 3:** Quality Outcomes of the Alberta Primary Care Endoscopy Study.

Physician	Colonoscopies Performed	Crude Proportion Successful Cecal Intubations (%)	Adjusted Proportion Successful Cecal Intubations (%)	USMSTF Adjusted Proportion Successful Cecal Intubations (%)^a^	Average Adenoma Detection^b^	Proportion Males^c^ ≥50years with ≥1 Adenoma (%)	Proportion Females^d^ ≥50years with ≥1 Adenoma (%)	Average Procedural Times (min)	Average Withdrawal Time^e^ (min)	Serious Adverse Events (#)
1	54	96.3	96.3	96.3	0.46	60.0	20.0	24.4	8.4	1
2	72	100	100	100	1.01	57.9	33.3	24.6	5.2	0
3	95	96.8	98.9	97.9	0.35	23.8	20.0	21.6	9.7	0
4	31	96.8	100	96.8	0.36	27.3	0.0^f^	20.7	4.9	0
5	38	89.5	94.4	91.9	0.13	25.0	0.0^g^	19.7	4.1	0
6	40	92.5	92.5	92.5	0.6	57.1	37.5	26.3	5.7	0
7	38	94.7	94.7	94.7	0.37	50.0	30.0	21.5	3.8	0
8	82	87.8	90.0	88.9	0.31	30.4	14.3	23.6	6.2	2
9	78	97.4	97.4	97.4	1.54	66.7	57.9	27.7	9.1	0
10	49	100	100	100	0.61	70.0	50.0	23.2	7.8	1
**Overall Means** *	**58 (SD = 22.3)**	**95.3 (93.3, 96.9)**	**96.5 (94.6, 97.8)**	**95.8 (94.1, 97.5)**	**0.62 (0.51, 0.74)**	**46.4 (38.5, 54.3)**	**30.2 (22.3, 38.2)**	**23.6 (22.7, 24.5)**	**7.0 (6.6, 7.4)**	**4**

a USMSTF adjusted proportion of successful cecal intubations excludes incomplete colonoscopies due to poor bowel preparation and severe colitis.

b Number of pathologically confirmed adenomas/number of colonoscopies ^c,d^ Proportion of males^c^ or females^d^ ≥50 years old, first time colonoscopy with pathologically confirmed adenoma

e Average withdrawal time of procedures where no lesions were detected

f Only 2 colonoscopies performed on this patient cohort;

g Only 3 colonoscopies performed on this patient cohort.

% = percent; min = minutes; # = number, serious adverse events (SAEs) reported as totals.

* Overall means reported with 95% confidence intervals except age reported with standard deviations.

#### Predictors of incomplete colonoscopies

The odds of having an incomplete colonoscopy were significantly increased in patients with poor bowel preparations, (OR = 4.5; 95% CI: 1.2–17.2) and patients over the age of 65 years (OR = 2.9; 95% CI: 1.3–6.3). Female patients were also at higher risk of having an incomplete colonoscopy, but this was not statistically significant (OR = 2.2; 95% CI: 0.97–5.15). The type of PCP endoscopist (family physician or general internist), volume of colonoscopies performed, indication for colonoscopy or inpatient status did not significantly influence the proportion of successful cecal intubations.

#### Adenoma detection

A total of 360 adenomas were pathologically confirmed (272 adenomas, 50 advanced adenomas, 34 serrated adenomas and four advanced serrated adenomas) for an overall average of 0.62 adenomas/colonoscopy. Individual physicians’ adenoma detection average ranged from 0.13 to 1.54 adenomas/colonoscopy. Although the participant with the shortest average withdrawal time also had the lowest average number of adenomas/colonoscopy, collectively the five physicians whose average withdrawal times were <6 minutes (mean withdraw time 4.7 min) had similar adenoma detection averages (0.58 vs. 0.65): p = 0.28 compared to the five who averaged ≥6 minutes withdrawal times (mean withdraw time 8.4 minutes).

At least one adenoma was found in 46.4% (95% CI: 38.5–54.3) of males and 30.2% (95% CI: 22.3–38.2) of females ≥50 years of age undergoing their first colonoscopy. These rates were greater than the USMSTF benchmarks of 25% and 15% (p<0.0001 for both). Twelve cases of colorectal cancer were pathologically confirmed for a colorectal cancer incidence of 2.1% (95% CI: 5.2, 18.7).

#### Potential serious adverse events

A total of 18 potential adverse events were investigated, nine reported by a patient and nine reported by a study physician. External adjudicators concluded that four serious adverse events (three bleeds and one perforation) occurred. All three bleeds occurred after snare cautery for advanced adenomas in patients >50 years of age. These three patients were admitted to hospital (median stay of 2 days). One patient was transfused 3 units of packed red blood cells, while no patient required a repeat colonoscopy or surgery. The perforation occurred during rectal retroflexion in a 79-year-old male with radiation proctitis. The complication was recognized during the procedure; the patient had a laparotomy with primary repair of the defect and was discharged home from his local hospital five days later.

The calculated risk of post-colonoscopy bleeding [3/577 = 0.52% (95% CI: 0.11, 1.5%)] was not significantly different than the target of ≤1/100 [33], (p = 0.40) and the perforation rate 1/577 = 0.17% (95% CI: 0.004%, 0.96%)] was between the accepted targets of 1/500 and 1/1000 [32], [33]. There were no serious complications related to procedural sedation and no deaths in the study.

#### Sedation agents and doses

Midazolam (Versed) was the most commonly used sedation agent (n = 570), followed by Fentanyl (n = 494) and propofol (Diprivan) (n = 140). Overall, only five physicians used propofol. Three endoscopists (two who were also general practice- anesthetists and one who had sedation administered by an anesthetist) accounted for 86.4% of the propofol use. One case was performed without any sedation.

#### Patient comfort level

Patients tolerated the colonoscopies, experiencing only one or two well-tolerated episodes of discomfort in 45.1% of the cases and no discomfort in 40.4% of cases.

#### Specialist referrals

Twenty-eight patients (4.8%; 95% CI: 3.2, 6.9]) were referred to a specialist for their gastrointestinal problems: 20 for definitive surgical management and eight for ongoing disease management.

#### Patient satisfaction survey

**Figure 1:**
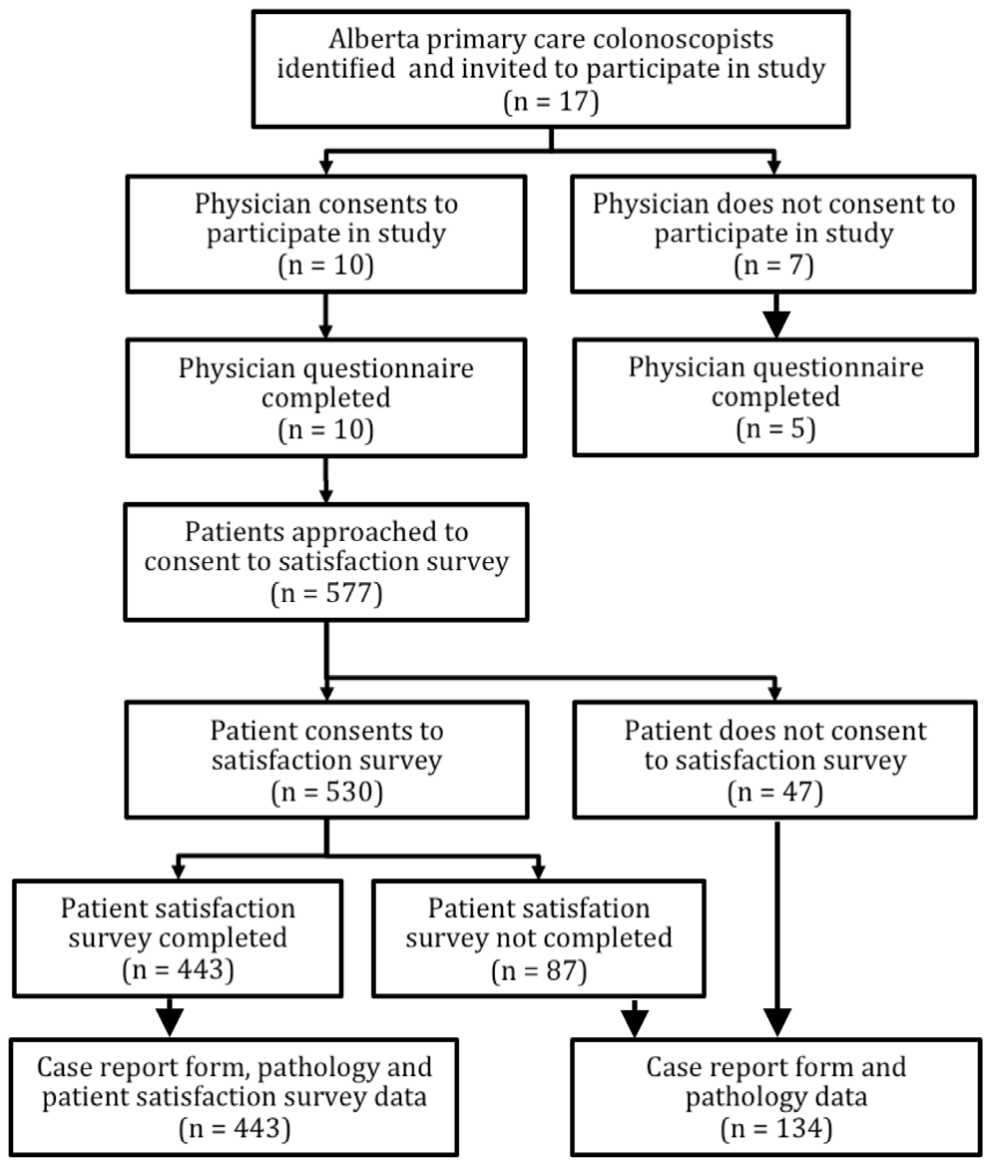
Alberta Primary Care Endoscopy Study Participation Results.

Five hundred and thirty of 577 (91.8%; 95% CI: 89.3–93.9) patients consented to the patient satisfaction phone survey, and 443 (83.5%) of the surveys were completed (Figure 1). Inability to contact the patient (55 cases) and withdrawal of consent (21 cases) were the most common reasons for not completing the satisfaction survey. Using 7-point Likert scales, the median wait time satisfaction score was 7 (IQR 5–7) and the median score for the hospital experience for their colonoscopy was also 7 (IQR 6–7). The overwhelming majority [440/443 (99.3%; 95%CI: 98.0–99.7)] of patients were willing to have a repeat colonoscopy performed by their primary care colonoscopist.

## Discussion

To our knowledge, this is the largest multi-centered PCP endoscopy study conducted in Canada, and the first prospective study with standard, robust, and externally adjudicated outcome measures. The APC-Endo Study demonstrated that PCPs can achieve standard benchmarks in colonoscopy performance. The group’s adjusted proportion of successful cecal intubations cecal intubations was 96.5%, with all physicians achieving adjusted cecal intubation targets of ≥90%. Our adjusted cecal intubation success formula differs from the USMSTF formula [32], [33] in that it reflects the skills of the primary care colonoscopist (do not dilate strictures) and does not include equipment malfunction, which is outside of the control of the endoscopist. Even using the USMSTF definition of adjusted cecal intubation success, the group achieved the benchmark standard of 90%.

These results compare favorably with published specialist data. For example, in a study of 17,868 colonoscopies, only 55% of American and Canadian gastroenterologists achieved cecal intubation rates ≥90% [39]. Another study of 5,477 colonoscopies performed by 10 American gastroenterologists revealed cecal intubation rates of 89.8% [40]. In Canada, the Practice Audit in Gastroenterology (PAGE) program’s provincial cecal intubation rates were between 86–94% [41], and another study of gastroenterologists and general surgeons found 10% had cecal intubation rates of <85% [8]. It is important to recognize that even specialists may have difficulties achieving cecal intubation targets.

The average number of adenomas detected per colonoscopy is a more meaningful marker of quality colonoscopy as it evaluates both technical competency, and indirectly measures appropriate patient selection, colonoscopy intervals, and bowel preparation quality. The APC-Endo Study PCPs detected on average 0.62 adenomas/colonoscopy, with individual physician adenoma detection ranging from 0.13 to 1.54 adenomas/colonoscopy. This variability may be partially explained by patient age and indication for the colonoscopy; however, existing literature demonstrates similar inter-physician variability in adenoma detection. In one study, the overall adenoma detection by 12 experienced gastroenterologists was 0.47 adenomas/colonoscopy, with individual physician’s results ranged from 0.1 to 1.05 adenomas/colonoscopy [42]. Another study of over 10, 000 colonoscopies performed by nine gastroenterologists concluded that the colonoscopist affects adenoma detection more than patient age or gender [43].

In our study, the proportion of males and females, over 50 years old, undergoing their first colonoscopy with at least one adenoma was 46.4% and 30.2% respectively. These results exceed the USPMTF minimum benchmarks of 25% and 15% [32], [33], but are comparable to rates observed in an Albertan colorectal cancer screening facility (Jonathon Love, University of Calgary, personal communication).

Serious adverse events were comparable to suggested targets and to results seen in large Canadian population-based studies. These studies report overall bleeding rates between 1.0 to 1.6/1000 colonoscopies [44], [45], which increase to 6.4/1000 in cases were polypectomies were performed [44]. While our observed bleeding risk of 5.2/1000 colonoscopies appears within these margins, the relatively small sample size provides wide confidence intervals. Our perforation rate is also comparable to results seen in clinical practice [44]–[46]. Recent Canadian studies report perforation rates ranging from 1/769 at a single academic centre in Alberta [46] to 1/833 at four hospitals in Winnipeg [44] to 1/1176 from a database involving colonoscopies in four provinces [45].

In our study, patients were highly satisfied with both their wait time for colonoscopy, and with their hospital experience during the colonoscopy. Previous studies demonstrate patient dissatisfaction with their wait time for a gastroenterologist consultation [47] and endoscopy [48]. Alberta has the longest gastrointestinal wait times of any province in Canada [9]. However, since wait time data were not collected and a control group was not identified, it remains unclear if wait times differences for consultation and endoscopy exist between PCP endoscopists and gastroenterologists.

Few patients were referred to specialists, implying that the study physicians are competent in managing their patients’ clinical symptoms and endoscopic findings. The majority of patients were referred for definitive surgical management of their gastrointestinal condition. This referral pattern (from primary care colonoscopist to surgeon) may improve patient flow through the health care system, and decrease the time between symptom onset and definitive care for diseases like colorectal cancer.

## Limitations

Only 10 of 17 Alberta primary care colonoscopists participated in the study. Practicing endoscopists who did not participate and less experienced colonoscopists may have different results. It is uncertain whether having both an endoscopy team member and the physician endoscopist complete the case report form eliminated all reporting bias, and as the physicians were aware of the study, the “Hawthorne Effect” may have inflated study outcomes. Furthermore, it is unclear whether the subset of patients who did not complete the satisfaction survey would have had similar patient satisfaction results as those who completed the survey. Finally, data collection occurred for only two months. While short evaluations of quality outcomes in endoscopy are common in Canada [8], [41], collecting and reporting on outcomes over a longer term would be a logical progression.

### Conclusions

The APC-Endo Study is the most comprehensive study to date to report on the quality of colonoscopies performed by a group of PCPs. Relatively experienced PCP colonoscopists can achieve quality benchmarks in cecal intubation and adenoma detection with a low complication rate and high patient satisfaction. Based on these results, training selected PCPs in gastrointestinal medicine and endoscopy should be encouraged to improve patient access and decrease endoscopic wait times, especially in rural settings.

## Supporting Information

Form S1 **APC Endo Case Report Form.**
(DOCX)

Form S2 **APC Endo Patient Satisfaction Survey.**
(DOCX)
