# Supplementary material for: Prospective Study of the Quality of Colonoscopies Performed by Primary Care Physicians: The Alberta Primary Care Endoscopy (APC-Endo) Study
Source: PLoS One. 2013 Jun 27;8(6):e67017. doi: 10.1371/journal.pone.0067017 (PMC3695091; doi:10.1371/journal.pone.0067017)
Supplement: Form S1 — APC Endo Case Report Form. (DOCX) [file pone.0067017.s001.docx]

**Alberta Primary Care Endoscopy Study (APC-Endo)**

**Case Report Form**

*****To be completed by endoscopy nurse and physician endoscopist*****

**Patient Code #: ________________**

Date __________________ Patient’s Age: ___________ Sex: 🞏 Male 🞏 Female Inpatient: 🞏Yes 🞏No

**Is this the patient’s first time having a colonoscopy?** 🞏 Yes 🞏 No

**Bowel preparation used**: 🞏 GoLytely (4 litres evening before) 🞏 GoLytely split prep 🞏 Pico-salax 🞏 Milk of Magnesia 🞏 Magnesium citrate 🞏 Oral fleet Phospho-soda 🞏 Other: ____________________________

**Predominant Indication: *(circle*** ***only one*** *– the most compelling reason for the colonoscopy)*

CRC Screen: Symptoms: Follow Up Scope: Other:

No FHx Pain/diarrhea/constipation IBD FU Abnormal x-ray

FOBT + Rectal Bleed CRC FU _________________

FHx + Anemia Polyp FU

HNPCC/FAP FHx

**Procedural Times:** *(24 hour clock, round to nearest minute)*

Procedure Start time *(scope insertion into anus)*: ___ ____:____ ___ Cecum identified: ___ ____:____ ___ Leaving Cecum: ___ ____:____ ___ Procedure End time: *(scope removed from anus)* ___ ____:____ ___

**Quality of Bowel preparation at end of procedure**: *(choose most appropriate response for entire colon)*

🞏 Excellent (no more than small bits of adherent feces / fluid)
🞏 Good (small amounts of feces or fluid, but not limiting exam)
🞏 Satisfactory (enough feces or fluid that may have limited the exam)
🞏 Poor (semi-solid waste could not be cleared resulting in poor views of mucosa)

**Cecal Intubation:**  🞏 Yes 🞏 No

If not, why: 🞏 Technically difficult 🞏 Poor bowel prep 🞏 Stricture 🞏 Equipment problem 🞏Other

**Cecal intubation verified by visualization of:** *(tick all that apply)*

🞏 Appendix 🞏 Trifold 🞏 Ileocecal valve 🞏 Intubation of t. ileum 🞏 Light in RLQ

Photo taken of cecal landmarks 🞏 Yes 🞏No

**Sedation used: (agents and amounts):** _________________________________________________________________________

**Patient comfort level during procedure**

🞏 No discomfort-resting comfortably throughout procedure
🞏 One or two episodes of discomfort, well tolerated
🞏 More than two episodes of discomfort adequately tolerated
🞏 Significant discomfort experienced several times during the procedure
🞏 Extreme discomfort experienced frequency throughout the procedure

**Alberta Primary Care Endoscopy Study (APC-Endo)**

**Polyp detection**

**Type of polyp / lesion**

**H** = hyperplastic

**A** = adenoma < 1 cm*

**AA** = adenoma > 1 cm*

**C** = cancer

**
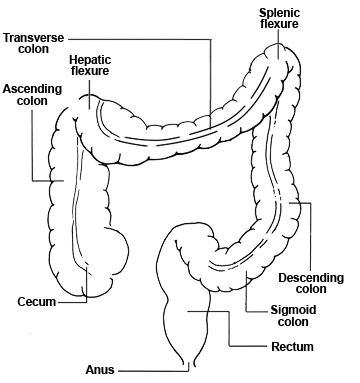
**

**Polypectomy Method**

1. Regular Biopsy
2. Hot biopsy forceps
3. Cold snare
4. Hot (cautery) snare

Using nomenclature in boxes, mark the location of ALL polyps found.

Mark **X** if the polyp was not retrieved.

**Largest diameter of polyp estimated by endoscopist using outer width of open biopsy forceps normally = 8mm*

**Predominant Finding:** *(circle the one most important clinical finding)*

Normal Colorectal Cancer

Adenoma polyp > 1 cm* Adenoma polyp < 1 cm* Hyperplastic polyp

IBD: old IBD: new Infectious colitis (incl. c. diff)

Diverticulosis** Hemorrhoids / Fissure** Other: ______________________________

** Largest diameter of polyp estimated by endoscopist using outer width of open biopsy forceps normally = 8 mm*

** *To be considered as predominant finding, must be consistent with presenting symptoms*

**Immediate complications:** *(see information sheet for definitions of complications)*

🞏 None 🞏 Bleeding 🞏 Perforation 🞏 Sedation 🞏 Other: _________________________

**Anticipated Referral to Specialist:** 🞏 Yes 🞏 No

If Yes, reason for referral: 🞏Surgery 🞏Disease management 🞏Repeat colonoscopy 🞏Other:_______________

**Additional Notes**: ______________________________________________________________________________________________________

­­_________________________________________________________________________________________________________________________________

Endoscopist: ____________________________ Nurse: ___________________________________

**Please fax the completed form to Dr. M. Kolber at (780) 407-3982**
