# Supplementary material for: Prospective Study of the Quality of Colonoscopies Performed by Primary Care Physicians: The Alberta Primary Care Endoscopy (APC-Endo) Study
Source: PLoS One. 2013 Jun 27;8(6):e67017. doi: 10.1371/journal.pone.0067017 (PMC3695091; doi:10.1371/journal.pone.0067017)
Supplement: Form S2 — APC Endo Patient Satisfaction Survey. (DOCX) [file pone.0067017.s002.docx]

**Alberta Primary Care Endoscopy Study (APC-Endo)**

**post procedure telephone SURVEY**

****Top section to be completed by endoscopy nurse or endoscopist****

**Patient Code #: _____________________**

Patient Consents to Participate in Post Procedure Telephone Survey: **🞏 Yes 🞏 No**

*If Yes, please complete the demographics section below and fax to Dr. M. Kolber*

*If No, please do not complete the demographics section, but still fax form to Dr. M. Kolber*

Patient Name _________________________________ Patient DOB ___________________ **Patient AHC # __________________________**

Procedure date _________________________ Endoscopist ___________________

Patient preferred phone # ________________________________ Preferred time to call: AM PM Evening

Patient alternate phone # ________________________________ Preferred time to call: AM PM Evening

**Please fax thIS form to Dr. M. Kolber at (780) 407-3982**

**post procedure telephone Survey**

**SHADED SECTION TO BE COMPLETED ONLY BY DR. M. KOLBER or DESIGNATE**

Date(s) attempted: ____________________________________ Date spoke to patient: __________________________________

**Patient reported Delayed Complications:** Yes No

Perforation Bleeding Other: ___________________________________________________________________________________

Complication Verified: Yes No

**Satisfaction Survey**

1. How would you rate your satisfaction with the wait to have your colonoscopy: Extremely Dissatisfied 1 2 3 4 5 6 7 Extremely satisfied

2. How would you rate the level of discomfort you experienced during the colonoscopy:

(0) No discomfort (1) Mild (2) Moderate (3) Severe (4) Extreme discomfort Do not remember

3. How would you rate your visit to the hospital for the colonoscopy: Poor 1 2 3 4 5 6 7 Excellent

4. Would you be willing to have a repeat colonoscopy performed by the same physician, if required? Yes No

5. Do you anticipate being referred to a specialist for your GI complaint? Yes No

6. If yes, for what reason? Surgery Management of disease Repeat colonoscopy Other

Notes: ________________________________________________________________________________________________________________________

Reviewer Name: ___________________________ Date: ________________________
